# Effects of Artificial Intelligence Recognition–Based Telerehabilitation on Exercise Capacity in Patients With Hypertension: Randomized Controlled Trial

**DOI:** 10.2196/81400

**Published:** 2026-01-13

**Authors:** Qiuru Yao, Baizhi Qiu, Longlong He, Qin Wang, Jihua Zou, Donghui Liang, Shuyang Wen, Yingchao Liu, Gege Li, Jinjing Hu, Huan Ma, Guozhi Huang, Qing Zeng

**Affiliations:** 1 Center of Rehabilitation Medicine Zhujiang Hospital, Southern Medical University Guangzhou China; 2 School of Nursing Southern Medical University Guangzhou China; 3 GuangDong Engineering Technology Research Center of Brain Function Assessment and Neuroregulation Rehabilitation Guangzhou China; 4 Institute of Exercise and Rehabilitation Science Zhujiang Hospital, Southern Medical University Guangzhou China; 5 Department of Clinical Medicine Xiamen Medical College Xiamen China; 6 School of Rehabilitation Sciences Southern Medical University Guangzhou China; 7 Department of Rehabilitation Sciences The Hong Kong Polytechnic University Hong Kong China (Hong Kong); 8 Department of Traditional Chinese Medicine Zhujiang Hospital, Southern Medical University Guangzhou China; 9 Department of Cardiology Guangdong Cardiovascular Institute Guangdong Provincial People's Hospital, Guangdong Academy of Medical Sciences, Southern Medical University Guangzhou China

**Keywords:** hypertension, artificial intelligence, telerehabilitation, lifestyle change, exercise habit formation, randomized controlled trial

## Abstract

**Background:**

Hypertension remains a major global health challenge, significantly increasing cardiovascular and all-cause mortality risks. While exercise therapy is effective, conventional approaches face limitations in accessibility and personalization, compromising adherence. Artificial intelligence (AI)–assisted remote rehabilitation enables real-time monitoring and personalized guidance, offering a promising alternative. Nevertheless, its clinical benefits and applicability require further systematic validation.

**Objective:**

This study aimed to evaluate the efficacy of an 8-week AI-assisted telerehabilitation program on improving exercise capacity and related health outcomes in patients with hypertension.

**Methods:**

This prospective, dual-arm, parallel, open-label, randomized controlled trial enrolled 62 patients with hypertension recruited via convenience sampling. Participants were adults aged between 18 and 75 years with a confirmed hypertension diagnosis who were excluded for severe cardiac complications, recent myocardial infarction, unstable angina, or physical disabilities preventing exercise. The participants were randomly assigned (1:1) to an intervention group that received AI-assisted remote rehabilitation plus routine health education, or a control group that received health education and conventional offline exercise guidance. The supervised exercise program included warm-up, cardiorespiratory endurance, strength resistance, balance, and flexibility training, followed by a cooldown. Sessions lasted between 30 and 50 minutes and were performed at least 3 times weekly for 8 weeks. Assessments at baseline and 8 weeks included the 6-minute walk test (6MWT), cardiopulmonary exercise testing (CPET), International Physical Activity Questionnaire (IPAQ), Short-Form Health Survey 12 (SF-12), Patient Health Questionnaire-9 (PHQ-9), Generalized Anxiety Disorder-7 (GAD-7), exercise self-efficacy, blood pressure (BP), body weight, handgrip strength, and other health-related indicators. The primary outcome was the change in 6-minute walk distance (6MWD). Data were analyzed according to the intention-to-treat principle.

**Results:**

Throughout the 8-week intervention period, no serious adverse events related to the AI-assisted telerehabilitation intervention occurred. After 8 weeks, the intervention group demonstrated significantly greater improvements than the control group in 6-minute walk distance (6MWD; adjusted mean difference 62.77, 95% CI 26.33-99.22; *P*=.002), systolic BP reduction (adjusted mean difference 4.11, 95% CI 0.11-8.28; *P*=.046), IPAQ score (adjusted mean difference 658.96, 95% CI 159.23-1158.69; *P*=.011), exercise self-efficacy score (adjusted mean difference 21.71, 95% CI 13.59-29.82; *P<*.001), total exercise time (adjusted mean difference 98.24, 95% CI 49.39-147.08; *P*=.001) peak oxygen uptake (peak VO_2_) (adjusted mean difference 3.39, 95% CI 0.49-6.29; *P*=.026), and peak oxygen uptake percent predicted (peak VO_2_%pred) (adjusted mean difference 11.58, 95% CI 2.06-21.10; *P*=.021).

**Conclusions:**

Compared with conventional exercise rehabilitation, AI-assisted remote rehabilitation was found to improve exercise capacity, boost regular physical activity and exercise self-efficacy, and aid in systolic BP control among patients with hypertension. This study positioned AI-assisted rehabilitation as a scalable and effective strategy for real-world hypertension management. It further contributes actionable guidance for developing effective home-based exercise strategies tailored to populations with hypertension.

**Trial Registration:**

Chinese Clinical Trial Registry ChiCTR2300076451; https://www.chictr.org.cn/showproj.html?proj=208353

## Introduction

Hypertension is a major public health concern with an enormous economic and social burden [1]. The World Health Statistics 2023 report by the World Health Organization (WHO) states that the worldwide prevalence of hypertension reached 33%, and it remains an upward trend in the context of global aging [2]. Given the high prevalence and severity of hypertension, there is an urgent need to implement effective, widely available, and sustainable strategies for its management.

Current interventions for hypertension are divided into pharmacological and nonpharmacological treatments. While pharmacological therapy effectively lowers blood pressure (BP) and remains the clinical mainstay [3,4], its benefits are often limited by poor adherence and the risk of BP rebound upon discontinuation, which elevates the risk of major cardiovascular events [5,6]. In contrast, nonpharmacological management focuses on lifestyle modifications, such as regular physical activity, dietary changes (eg, salt intake restriction), and weight management. The health benefits of exercise are well-established—regular moderate-to-high intensity aerobic activity can reduce BP by an average of 11/5 mm Hg [7]. Furthermore, its efficacy in lowering BP may be comparable to that of pharmacological interventions [8].

Regular exercise, a key nonpharmacological intervention, significantly reduces hypertension risk, improves BP control, and plays a vital role in its prevention and management [9]. Regular exercise not only significantly lowers BP but also helps regulate weight, improve lipid and blood sugar levels, and promote overall metabolic function. Engaging in moderate physical exercise before the onset of hypertension, combined with a healthy lifestyle, can reduce the incidence of primary hypertension by 54% [10]. Moderate exercise can help delay the progression of hypertension, while excessive exercise may cause BP to rise, thereby increasing the risk of cardiovascular and cerebrovascular diseases. However, the current state of physical activity in patients with hypertension is not encouraging. Patients with hypertension report being less physically active compared with individuals without hypertension, and they are less likely to meet physical activity recommendations [11]. Furthermore, they often encounter numerous obstacles when it comes to maintaining regular physical activity, such as a lack of self-efficacy, social support, and adequate supervision [12]. Therefore, selecting appropriate exercise methods and training approaches for patients with hypertension is of great significance for their BP management [13,14].

The rapid development of digital therapeutics in recent years has the potential to be an important part of BP management in the future [15]. It is an emerging, promising field of medicine that aims to implement lifestyle changes and ultimately facilitate disease management using software programs such as smartphone applications and device algorithms [16,17]. Digital therapeutics are gaining ground in the fields of medicine and health care, and the HERB Digital Hypertension 1 (HERB-DH1) pivotal trial conducted in Japan is one such success case [18]. Although there are many mobile technologies available for improving BP management, unfortunately, only a few of them have been developed with the involvement of health care professionals or medical organizations, and the evidence of their long-term benefits in terms of BP management is still scarce [19,20]. Meanwhile, existing digital therapeutics (such as the HERB-DH1 behavioral algorithm) face limitations due to the absence of real-time motion feedback mechanisms, making it challenging to effectively monitor and ensure patient adherence to exercise prescriptions. This technological gap highlights the medical potential of 3D skeletal pose recognition technology. By capturing and analyzing human motion data, this technology enables real-time corrective guidance and dynamically adaptive interventions, serving as an intelligent monitor that safeguards exercise safety and efficacy.

Among numerous artificial intelligence (AI) technologies, posture recognition, as a technology capable of understanding and interpreting human behavior, is gradually becoming a hot topic of research. As an important branch of AI, posture recognition technology enables machines to better understand human behavior by capturing, analyzing, and interpreting human movements, thereby achieving a leap forward in human-machine interaction. Posture recognition technology detects the position and orientation of a person or object from a video or image and can significantly improve the accuracy and quality of telerehabilitation services [21]. Human posture estimation techniques use input data, such as images, to help with reconstructing human representations to assist in measuring changes in patient movement and assessing limb or joint function [22] and to provide feedback during the treatment process to help patients understand and correct postural errors. Research has shown [23] that an AI-based telerehabilitation system, combined with 3D posture recognition technology, can be a viable alternative to exercise intervention for patients with sarcopenia. In our previous work in the field of low back pain using AI-assisted telerehabilitation technology, we found that AI-assisted multimodal movement-based telerehabilitation could significantly improve patients’ lower back pain and provide better therapeutic outcomes than traditional movement-based telerehabilitation [24]. This technology significantly improves the accuracy of training guidance through high-precision motion capture and real-time feedback mechanisms, and its intervention effect is equivalent to that of traditional face-to-face training and conventional telerehabilitation programs, while optimizing the quality of standardized execution of rehabilitation training. However, there is insufficient evidence in the field of hypertension.

Considering the above, we utilized a novel smartphone app, developed under the guidance of health care professionals, to address this need. This app was designed to help patients with hypertension develop a habit of regular physical activity and improve their overall quality of life. Its advantage lies in providing online, personalized exercise prescriptions, thereby enabling a scientific, effective, and targeted remote training intervention. Accordingly, this study aimed to examine the effects of this AI-assisted remote rehabilitation system on exercise capacity, self-efficacy, psychological well-being, and related health indicators in patients with hypertension. We hypothesized that this intervention would positively influence these treatment outcomes compared to usual care.

## Methods

### Study Design

This study was a multicenter, open-label randomized controlled trial conducted in Guangdong Provincial People's Hospital (Guangzhou, China) and Zhujiang Hospital, Southern Medical University (Guangzhou, China), 2 large tertiary care hospitals in southern China. The reporting of this trial conformed to the CONSORT (Consolidated Standards of Reporting Trials) guidelines for randomized controlled trials, and the study protocol adhered to the SPIRIT (Standard Protocol Items: Recommendations for Interventional Trials) checklist (Multimedia Appendix 1) [25,26]. The study was prospectively registered in the Chinese Clinical Trial Registry (ChiCTR2300076451) on October 9, 2023.

### Ethical Considerations

#### Overview

This study was conducted in accordance with the principles of the Declaration of Helsinki and relevant Chinese regulations governing clinical research. The study protocol was reviewed and approved by the ethics committee of Zhujiang Hospital, Southern Medical University (2023-KY-190-01). The study was also filed in the Medical Research Registration Information System of the National Health Commission of China. All participants provided written informed consent prior to enrollment. The consent form detailed the study objectives, procedures, potential risks and benefits, and participants’ rights. For participants who were unable to continue the study due to health or personal reasons, data were retained and analyzed in accordance with the intention-to-treat principle, as approved by the ethics committee.

#### Privacy and Confidentiality

All participant data were deidentified prior to analysis. Personal information and medical records were stored securely and accessible only to authorized research personnel. Specifically, for the AI-assisted telerehabilitation, the technology platform was designed to safeguard privacy by not recording any personal identifiers (eg, real names or ID numbers). Unless users explicitly opted in for video recording, the system did not capture or store any recognizable video footage. Instead, it processed the video stream in real time to extract only the coordinate data of 17 skeletal key points, using this anonymized information to compute exercise completion and quality scores. Data anonymity was maintained throughout the study and in all publications.

#### Compensation

Participants did not receive financial compensation for their involvement in the study. However, they were provided with free health education materials and access to the AI-based telerehabilitation application during the intervention period.

#### Use of Images and Identifiable Information

The images included in this paper (eg, app interfaces, training scenarios) do not contain any identifiable images of the participants. All screenshots and illustrations are system display interfaces and have been obtained with the consent of the system owner and the person who took the photos. Therefore, no additional consent for image publication was required.

### Participant Selection and Recruitment

Recruitment was conducted from November 2023 to October 2024. Given the pragmatic nature of this trial and the need to consecutively enroll eligible patients, a convenience sampling method was employed. Participants were recruited from the cardiology outpatient clinic and via community advertisements. Participants were primarily recruited from the cardiology outpatient clinics of Guangdong Provincial People's Hospital (Guangzhou, China) and Zhujiang Hospital, Southern Medical University (Guangzhou, China), and through community advertisements in the surrounding areas.

Recruitment information was disseminated through a combination of online and traditional channels. Online strategies included utilizing social media platforms, such as WeChat, to publish recruitment announcements and conduct preliminary online screenings. Traditional methods involved on-site activities (eg, setting up recruitment booths in hospitals and communities to provide briefings and answer questions directly), physician referrals from established partnerships, and the distribution of printed leaflets and posters.

### Inclusion and Exclusion Criteria

#### Inclusion Criteria

The inclusion criteria were as follows: age 18 to 75 years; diagnosis of essential hypertension, defined as an office systolic blood pressure (SBP) ≥140 mmHg and/or diastolic blood pressure (DBP) ≥90 mmHg; no cognitive impairment or mental illness; passed the physical activity readiness questionnaire screening or approved by a physician prior to participation; ability to communicate normally and cooperate with the questionnaire survey; not participating in other research involving healthy lifestyle promotion, particularly physical exercise; no prior exercise habit, defined as exercising less than 3 times per week for under 30 minutes per session; and ownership of a smartphone with access to WeChat and the internet.

#### Exclusion Criteria

The exclusion criteria were as follows: patients with severe or ineffectively controlled hypertension (SBP ≥180 mmHg and/or DBP ≥110 mmHg at rest); patients with pulmonary hypertension, severe hypertension, hypertensive crisis, unstable stage III hypertension, hypertensive encephalopathy, acute hypertension, or other serious complications such as severe arrhythmia, tachycardia, heart failure, unstable angina pectoris, or obvious adverse reactions to antihypertensive drugs with failure to control; patients with malignant tumors or hypertension combined with left ventricular ejection fraction <55%, aortic, mitral, tricuspid, or pulmonary stenosis, severe mitral, tricuspid, or pulmonary valve insufficiency, hypertrophic cardiomyopathy, acute infection, fundus hemorrhage, diabetic acidosis, gangrene of the lower limbs, severe hypothyroidism, or renal insufficiency; patients with motor organ injury who underwent bone or joint surgery within the past year, such as hip or knee replacement or spine surgery; patients unable to understand or answer questions or complete questionnaires due to subjective or objective reasons; and participants considered unsuitable for clinical trials by the researchers.

### Sample Size

In this study, the sample size was calculated using the 6-minute walk test (6MWT) distance as the primary outcome indicator. According to the relevant literature [27,28], telemedicine has been shown to improve exercise capacity and physical function in patients with cardiovascular disease. Setting the minimal clinical difference significance (>33 m), 2-sided α=.05, and the test power 1-β 0.90. Calculations were performed using PASS software (version 15.0; NCSS LLC). Considering the loss and refusal rate of 20%, a minimum of 29 participants for each group was required, for a total of at least 58 participants.

### Randomization and Blinding

Block randomization was performed using SAS software (version 9.3; SAS Institute Inc) to ensure balanced group sizes and prevent the potential for imbalance inherent in simple randomization. The randomization process allocated participants to either the intervention or control group using a block size of 6, across a total of 10 blocks, to generate the random sequence and obtain group allocations. The allocation sequence was concealed using opaque, sealed envelopes. All researchers responsible for patient recruitment and data collection were blinded to the sequence generation and were not involved in preparing the concealment envelopes.

### Interventions

#### Remote Exercise Rehabilitation

**Figure 1:**
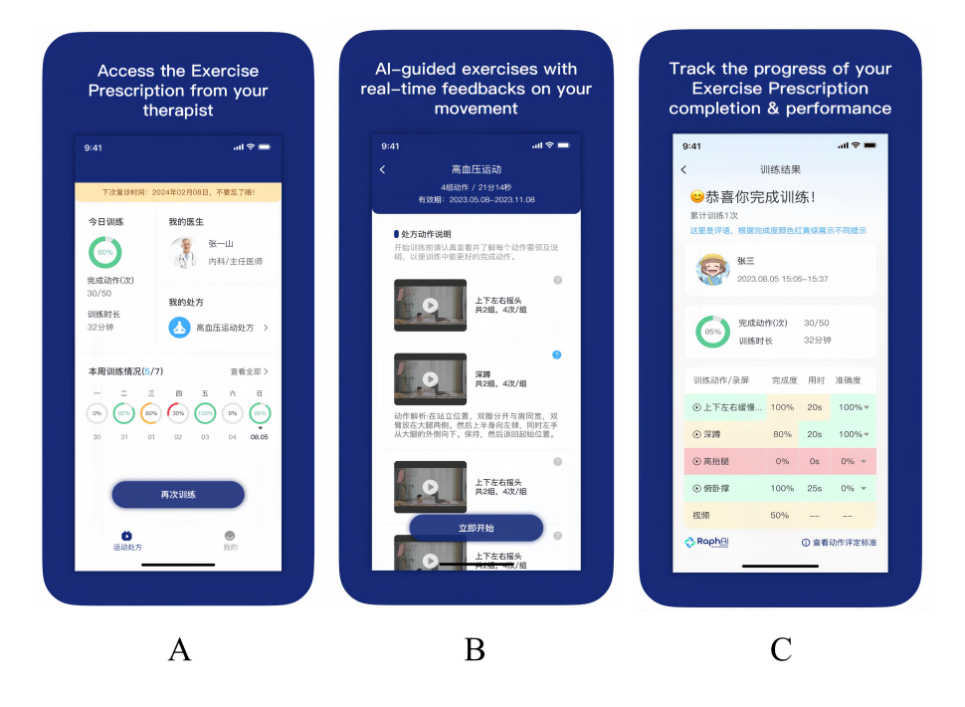
Interface demonstration of the “Rehabilitation Exercise Prescription” mobile app used for artificial intelligence (AI)–assisted telerehabilitation. A: main landing page of the app; B: interface showing a personalized exercise prescription generated for a participant; C: screen displayed upon completion of an exercise session, summarizing performance metrics.

Based on hypertension health education and exercise guidance, the intervention group used the “rehabilitation exercise prescription” App (RaphAI Health Technologies Limited) to carry out AI-assisted remote rehabilitation exercise intervention (Figure 1). The specific procedures included are detailed as follows.

#### Assessment

Medical history was collected using the online assessment questionnaire, and each patient’s general condition was evaluated through a combination of physical examination and questionnaire results. The assessment included (1) general information, including basic information (name, gender, age, contact information), behavioral data (smoking, drinking habits, eating habits), and medical history (past medical history, history of hypertension treatment, family history of hypertension, BP measurement); (2) current physical activity level (regular exercise, type and frequency of exercise); (3) anthropometric data (height, weight, waist circumference, and hip circumference); (4) the American Heart Association (AHA)/ American College of Sports Medicine (ACSM) Health/Physical Fitness Preexercise Screening Questionnaire; (5) physical fitness assessments (cardiorespiratory endurance, strength, coordination, flexibility and balance assessment); and the (6) Physical Activity Readiness questionnaire (PAR-Q).

#### Exercise Prescription Generation and Delivery

For the prescription generation, 4 personalized cardiopulmonary rehabilitation prescriptions were customized according to the standards of the ACSM. After the assessment, the system determined the patient's risk stratification according to the self-assessment and offline assessment results and automatically generated exercise prescriptions.

The prescription was pushed to the patient 5 times a week for 30 to 50 minutes each time on a regular basis and was required to be completed at least 3 times a week. The content included warm-up, cardiopulmonary endurance training, strength resistance training, balance training, flexibility training, and cooldown.

#### AI Technology and Motion Capture Framework

The “Rehabilitation Exercise Prescription” app was built upon an AI engine that integrated TensorFlow, an open-source machine learning library, with the OpenPose architecture for real-time human pose estimation. This system employed a bottom-up approach using Part Affinity Fields to detect 17 key body joints (including eyes, nose, ears, shoulders, elbows, wrists, hips, knees, and ankles) and assembled them into a full-body skeleton without the need for physical markers. By processing video frames from the smartphone camera, the system tracked the coordinate changes of these skeletal key points across consecutive frames to quantify and analyze patient movement in real time.

#### Rehabilitation Training

**Figure 2:**
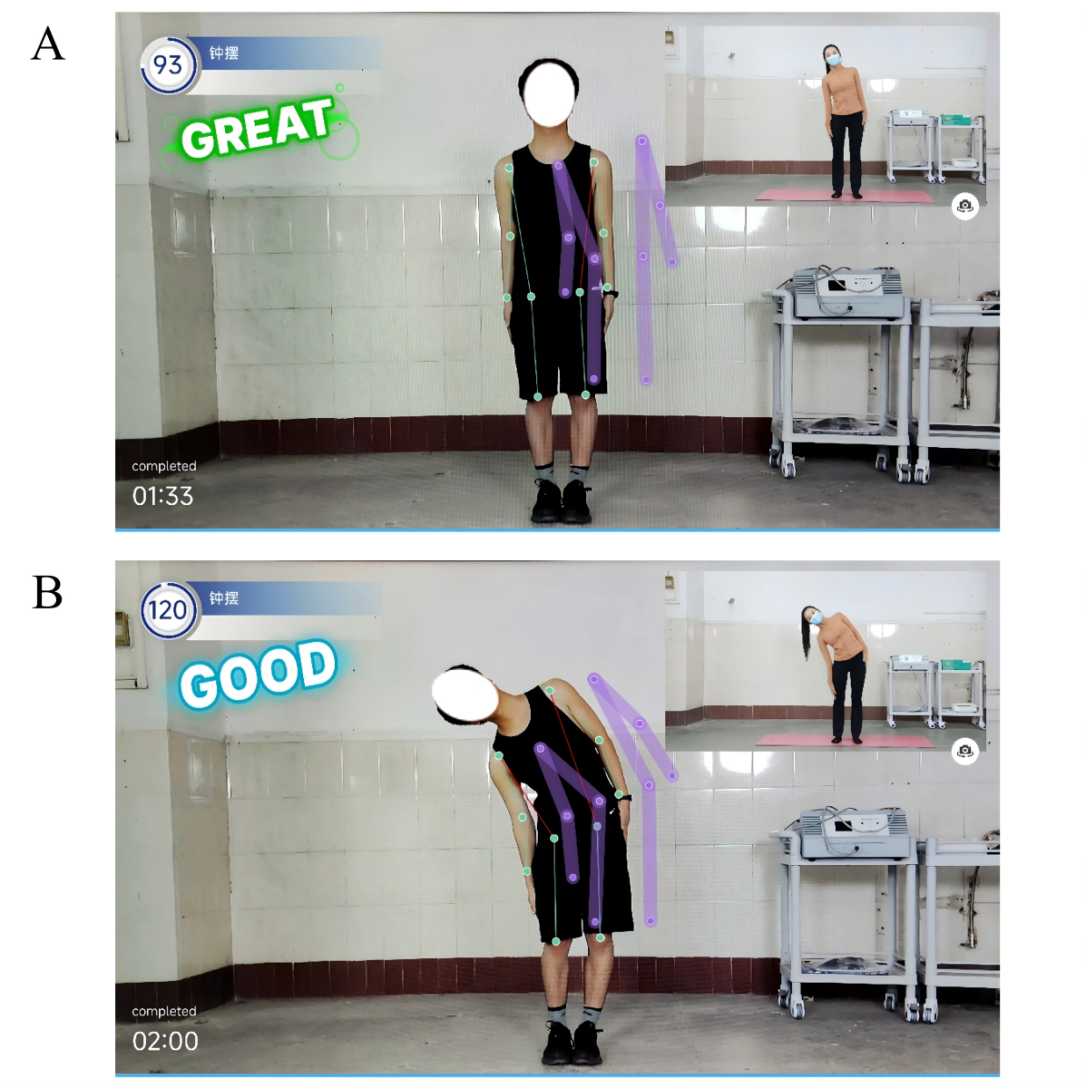
Examples of an artificial intelligence (AI)–assisted telerehabilitation process using the “Rehabilitation Exercise Prescription” app in patients with hypertension. A: scene during participant training; B: mobile interface showing real-time video and AI movement guidance.

The patients were trained according to the exercise prescription pushed by the app, and the action explanation was provided during the training. Computer AI visual recognition technology was used to identify 17 key bone points in the camera in a markerless way to monitor the training data in real time, calculate the angle and displacement of different parts of the body in real time, and provide intelligent feedback and corrective guidance (Figure 2).

#### Follow-Up and Optimization

**Figure 3:**
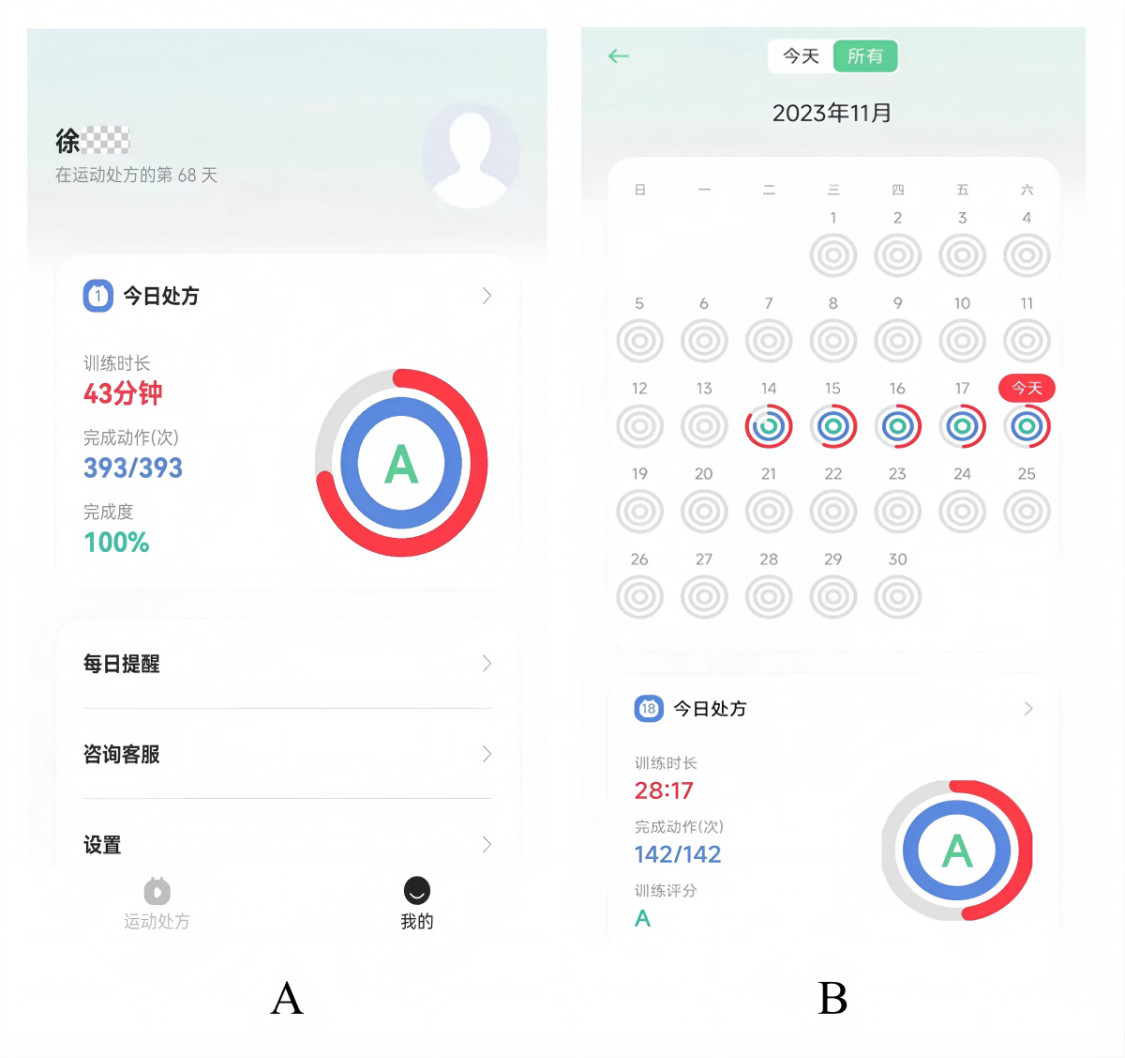
Postexercise evaluation and records via the “Rehabilitation Exercise Prescription” app. A: automatically generated assessment reports at the end of exercise; B: automated exercise diary recording interface.

After each training, the system automatically sent the perceptual experience score and the training report. The report included the overall score (three grades: A, B, and C), completion degree, accuracy, and time for each action, and generated an exercise diary. To enhance adherence, the system also incorporated an automated reminder function that pushed notifications to prompt users to initiate their scheduled training sessions (Figure 3).

We provided immediate consultation and guidance to participants through online dialogue platforms such as WeChat or telephone follow-up, answered participants’ questions during the exercise process, and adjusted the training program to suit each patient’s actual situation. In addition to in-program exercise training, the suitability of higher intensity exercise for participants depended on patient feedback, remotely monitored exercise performance data, and the results of weekly online and telephone consultation feedback from the participants. The exercise program could also be adjusted, and specific exercises could be changed for individual participants if the current exercise intensity could not be achieved.

We conducted weekly online consultations or telephone follow-ups to quantify follow-up data and optimize exercise prescriptions. If the participants were unable to adapt to the current intensity, the exercise program was adjusted and replaced with a specific exercise.

### Control Group

Participants in the control group received routine verbal health education on hypertension based on the current guidelines. This covered lifestyle guidance for hypertension, including dietary education emphasizing low-salt diets, balanced nutrition, alcohol restriction, adequate hydration, weight management, healthy food choices, psychological adjustment, smoking cessation guidance, sleep management, and advice on reducing disease risk and understanding medication. They also received exercise prescriptions, including warm-up, aerobics, strength, balance, and flexibility training types, intensity, and frequency, from the same movement library as the trial group. The prescription was 30 to 50 minutes per session, and patients were advised to complete it 5 times per week and required to complete it at least 3 times per week. Upon enrollment, the research team distributed the Hypertension Rehabilitation Handbook to all participants. This handbook was developed based on relevant guidelines [29-33] and covers the following topics: the definition and classification of hypertension, common misconceptions, prevalence statistics, explanations of hazards, risk factor analysis, identification of susceptible populations, methods of early detection and diagnosis, techniques for self-BP measurement, prevention and treatment guidelines, recommendations for lifestyle adjustments, guidance on exercise rehabilitation, and guidance on medication. During the study period, control group participants only received necessary BP measurements and safety assessments at follow-up points, with no active exercise supervision or feedback on execution quality.

### Statistical Analyses

All analyses were performed using SPSS statistical software (version 26.0; IBM Corp). Analyses were conducted according to the intention-to-treat principle and according to the intervention group to which participants were originally assigned, regardless of adherence to the intervention. Continuous variables are described as mean (SD) or median (IQR), and categorical variables are described as numbers (percentages). The baseline characteristics between groups were compared using independent *t* tests, Mann-Whitney U tests, or chi-square tests, as appropriate.

Missing data were handled using multiple imputations. The mechanism of missingness was evaluated using the missing completely at random (MCAR) test. The imputation procedure was performed using the fully conditional specification method, and we generated 50 imputed data sets to ensure efficiency and stability. The imputation model included all primary and secondary outcome variables, the treatment group, and auxiliary variables (eg, age and sex).

Between-group differences in primary and secondary outcomes were assessed using analysis of covariance (ANCOVA), with the 8-week measurement as the dependent variable and the baseline value as a covariate. Primary analysis was conducted on each of the 50 imputed data sets. The results were subsequently pooled to generate the final estimates, along with their 95% CIs and *P* values. The significance level was set at *P*<.05 for all statistical tests.

## Results

### Study Population

**Figure 4:**
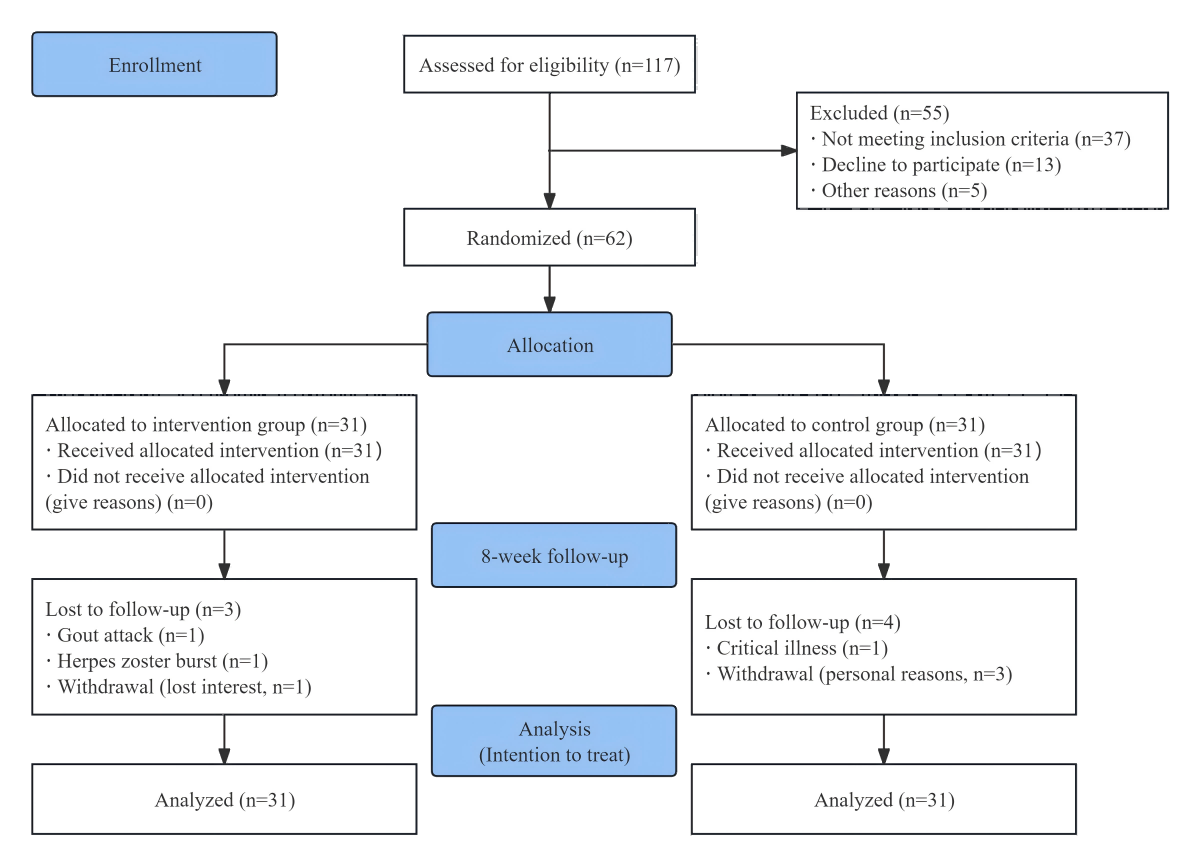
Flowchart of the study process.

A total of 62 participants were included in this study, 31 each in the intervention and control groups. During the follow-up period, 2 people in the intervention group discontinued exercise intervention due to sudden illness, 1 person was unable to exercise for personal reasons, 1 person in the control group was unable to exercise for physical reasons, and 3 people were unable to adhere to exercise for personal reasons. Finally, according to the principle of intention-to-treat analysis, 31 people in the intervention group and 31 people in the control group were included (Figure 4).

The average age of the participants overall was 52.95 (SD 11.46) years old. The average age of the intervention group was 50.48 (SD 9.44) years, and the average age of the control group was 55.42 (SD 12.86) years; there was no statistically significant difference between the 2 groups. Males accounted for 58% (n=36) and females accounted for 41.9% (n=26) of the participants. The average SBP was 128.87 (SD 12) mmHg, and the average DBP was 85.02 (SD 8.82) mmHg. The mean duration of hypertension was 7.14 (SD 7.5) years. The classification of hypertension was mainly grade 1 hypertension, accounting for 88.7% (n=55) of cases. The proportions of low-intensity, moderate-intensity, and high-intensity physical activity were 25.8% (n=16), 61.3% (n=38), and 12.9% (n=8), respectively. The results showed that the baseline data of the 2 groups were balanced, and there were no statistically significant differences (Table 1).

**Table 1:** Baseline characteristics of patients with hypertension in the artificial intelligence (AI)-assisted telerehabilitation randomized controlled trial (N=62).

Characteristic		All participants (n=62)	Control group (n=31)	Intervention group (n=31)	*P* value
Age (years), mean (SD)		52.95 (11.46)	55.42 (12.86)	50.48 (9.44)	.09
**Gender, n (%)**					.61
	Male	36 (58)	17 (54.8)	19 (61.3)	
	Female	26 (41.9)	14 (45.1)	12 (38.7)	
Height (m), mean (SD)		1.64 (0.07)	1.64 (0.09)	1.64 (0.06)	.77
Weight (kg), mean (SD)		65.42 (13.06)	66.32 (14.54)	64.53 (11.57)	.59
BMI (kg/m^2^), mean (SD)		24.03 (3.35)	24.21 (3.37)	23.86 (3.40)	.68
**Education, n (%)**					.82
	Primary and below	4 (6.5)	2 (6.5)	2 (6.5)	
	Junior high school	9 (14.5)	3 (9.7)	6 (19.4)	
	High school/technical secondary school	13 (21)	8 (25.8)	5 (16.1)	
	College/bachelor’s degree or above	36 (58.1)	18 (58.1)	18 (58.1)	
**Smoking history, n (%)**					.72
	Yes	9 (14.5)	5 (16.1)	4 (12.9)	
	No	53 (85.5)	26 (83.9)	27 (87.1)	
**Drinking history, n (%)**					.74
	Yes	11 (17.7)	5 (16.1)	6 (19.4)	
	No	51 (82.3)	26 (83.9)	25 (80.6)	
**Operation history, n (%)**					.59
	Yes	42 (67.7)	22 (71)	20 (64.5)	
	No	20 (32.3)	9 (29)	11 (35.5)	
Systolic BP^a^ (mm/Hg), mean (SD)		128.87 (12)	129.48 (14.32)	128.26 (9.33)	.69
Diastolic BP (mm/Hg), mean (SD)		85.02 (8.82)	853 (9.60)	85 (8.13)	.99
Amount of medication, mean (SD)		2.73 (1.57)	2.62 (1.66)	2.85 (1.51)	.73
Number of comorbidities, mean (SD)		1.98 (0.96)	1.93 (0.92)	2.04 (1.02)	.74
Duration of hypertension (years), mean (SD)		7.14 (7.50)	6.57 (7.51)	7.80 (7.63)	.52
**Grading of hypertension, n (%)**					.14
	1	55 (88.7)	28 (90.3)	27 (87.1)	
	2	5 (8)	1 (3.2)	4 (12.9)	
	3	2 (3.2)	2 (6.5)	0 (0)	
**IPAQ^b^ physical activity level, n (%)**					.20
	Low	16 (25.8)	7 (22.6)	9 (29)	
	Middle	38 (61.3)	22 (71)	16 (51.6)	
	High	8 (12.9)	2 (6.5)	6 (19.4)	

^b^BP: blood pressure.

^c^IPAQ: International Physical Activity Questionnaire.

### Data Availability and Handling of Missing Data

The extent of missing data at the 8-week follow-up was detailed in Tables S1 and S2 in Multimedia Appendix 2. The proportion of missingness for the primary outcome, 6-minute walk distance (6MWD), was 6.5% (4/62). For secondary outcomes, the missingness ranged from 0% to 14.5%. A dedicated subsample of 24 participants underwent cardiopulmonary exercise testing (CPET), among whom the missing rate for all CPET indexes was 12.5% (3/24; Table S2 in Multimedia Appendix 2). The MCAR test was performed on the data set for the entire cohort (χ²_596_ = 557.85; *P*=.87) and separately on the CPET (χ²_12_=0; *P*>.99). Both results were statistically nonsignificant, which meant that the data were missing completely at random. Therefore, multiple imputation was deemed appropriate and employed to handle missing values in the primary analyses.

### Comparison of Primary and Secondary Outcomes

There were no significant differences in waist circumference, hip circumference, grip strength, 6MWD, anxiety and depression, quality of life, and exercise self-efficacy scores between the 2 groups at baseline, indicating that the baseline levels of the indicators were comparable between the 2 groups before intervention.

The difference results showed that after the intervention, there were statistically significant differences in 6MWD (adjusted mean difference 62.77, 95% CI 26.33-99.22; *P*=.002), SBP reduction (adjusted mean difference 4.11, 95% CI 0.11-8.28, *P*=.046), IPAQ physical activity level (adjusted mean difference 658.96, 95% CI 159.23-1158.69; *P*=.011), exercise self-efficacy score (adjusted mean difference 21.71, 95% CI 13.59 to 29.82; *P*＜.001), between the 2 groups (Table 2).

**Table 2:** Comparison of primary and secondary health outcomes before and after the 8-week intervention in patients with hypertension.

Outcome	Intervention group (n=31)		Control group (n=31)			Between-group difference at 8 weeks		*P* value^a^
	Before (week 0)	After (week 8)	Before (week 0)	After (week 8)	Adjusted mean difference (95% CI)			
6MWD^b^ (m), mean (SD)	472.05 (79.58)	528 (69.54)	448.57 (138.53)	449.43 (94.60)	62.77 (26.33-99.22)		*.002* ^c^	
IPAQ^d^ (MET^e^-min/week), median (IQR)	1386 (510-2190)	1983 (1597.25-3037.50)	897 (636-1386)	1464 (1016.7-51924.50)	658.96 (159.23-1158.69)		.01	
PHQ-9^f^, mean (SD)	6.03 (3.91)	2.72 (3.31)	5.13 (4.49)	2.75 (1.82)	–0.14 (–1.64 to 1.36)		.85	
GAD-7^g^, mean (SD)	6.23 (4.38)	2.41 (2.20)	4.48 (4.21)	2.25 (1.68)	–0.23 (–1.26 to 0.80)		.65	
SF-12^h^, mean (SD)								
PCS^i^, mean (SD)	41.38 (6.86)	47.51 (6.12)	42.64 (9.06)	47.26 (6.65)	0.66 (–2.64 to 3.96)		.69	
MCS^j^, mean (SD)	46.28 (10.17)	54.37 (5.20)	46.34 (10.16)	54.18 (5.04)	0.19 (–2.68 to 3.07)		.89	
Exercise self-efficacy, mean (SD)	52.96 (30.02)	84.23 (15.84)	60.93 (28.37)	62.85 (12.67)	21.71 (13.59-29.82)		＜.001	
Weight (kg), mean (SD)	64.53 (11.57)	63.92 (11.04)	66.32 (14.54)	66.66 (14.27)	–1.02 (–1.96 to 0.08)		.053	
Girth (cm)	86.68 (9.76)	86.84 (9.80)	88.03 (9.31)	88 (9.40)	0.12 (–1.54 to 1.78)		.88	
Hipline (cm)	97 (8.03)	96.48 (7.61)	98.45 (8.16)	97.71 (8.43)	–0.06 (–2.49 to 2.37)		.96	
Right grip strength (kg), mean (SD)	32.87 (10.68)	35.89 (11.23)	26.49 (6.25)	27.70 (8.81)	4.39 (–4.67 to 13.44)		.33	
Left grip strength (kg), mean (SD)	29.68 (10.16)	34 (11.24)	25.71 (6.27)	26.73 (9.71)	4.85 (–3.68 to 13.38)		.25	
Systolic BP^k^ (mm/Hg)	128.30 (9.28)	116.63 (7.64)	129.96 (15.21)	120.92 (7.15)	4.11 (0.11-8.28)		.046	
Diastolic BP (mm/Hg)	84.52 (8.24)	75.56 (8.79)	85.25 (9.29)	77.71 (9.16)	1.81 (–2.72 to 6.35)		.43	

^a^The significance level was set at *P*<.05.

^b^6MWD: 6-minute walk distance.

^c^The italicized values are the *P* value of the primary outcome.

^d^IPAQ: International Physical Activity Questionnaire.

^e^MET: metabolic equivalent of task.

^f^PHQ-9: Patient Health Questionnaire-9.

^g^GAD-7: Generalized Anxiety Disorder-7.

^h^SF-12: Short-Form Health Survey 12.

^i^PCS: Physical Component Summary.

^j^MCS: Mental Component Summary.

^k^BP: blood pressure.

After the intervention, the comparison of cardiopulmonary exercise test indexes between the 2 groups, total exercise time (adjusted mean difference 98.24, 95% CI 49.39-147.08; *P*=.001)，peak oxygen uptake (peak VO2) (adjusted mean difference 3.39, 95% CI 0.49-6.29; *P*=.026) and peak oxygen uptake percent predicted (peak VO2%pred) (adjusted mean difference 11.58, 95% CI 2.06-21.10; *P*=.021) were statistically significant, and the exercise time of the intervention group was significantly longer than that of the control group. There was no significant difference in Watt, respiratory exchange rate, anaerobic threshold, anaerobic threshold %pred, HR rest, and HR peak between the two groups (Table 3).

**Table 3:** Comparison of cardiopulmonary exercise test (CPET) indices at baseline and after the 8-week intervention for both study subsample groups (N=24).

Outcome	Intervention group (n=12)		Control group (n=12)		Between-group difference at 8 weeks	*P* value^a^
	Before week 0), mean (SD)	After (week 8), mean (SD)	Before (week 0), mean (SD)	After (week 8), mean (SD)	Adjusted mean difference (95% CI)	
Exercise time (s)	472.17 (89.47)	564.29 (71.38)	481.67 (110.11)	457.63 (37.37)	98.24 (49.39-147.08)	.001
Maximum load (Watt)	112.17 (35.51)	131.71 (34.63)	97.33 (35.71)	93.35 (45.76)	17.08 (–18.29 to 52.44)	.31
Maximum load %pred	74.50 (11.29)	88.57 (8.42)	78.17 (19.72)	81.75 (12.43)	3.75 (–3.77 to 11.27)	.30
RER^b^	1.20 (0.13)	1.22 (0.09)	1.18 (0.10)	1.22 (0.11)	0.00 (–0.12 to 0.12)	.98
Peak VO_2_^c^ (ml/min/kg)	21.50 (4.80)	23.80 (2.89)	19.98 (4.44)	19.56 (4.35)	3.39 (0.49-6.29)	.03
Peak VO_2_%pred^d^	66.75 (9.78)	82.57 (5.80)	76.25 (15.47)	73.25 (14.96)	11.58 (2.06-21.10)	.02
Anaerobic threshold (ml/min/kg)	13.63 (3.01)	14.13 (2.03)	12.70 (2.48)	12.60 (3.31)	1.54 (–1.08 to 4.16)	.23
Anaerobic threshold%pred	42.08 (7.35)	49.86 (7.90)	48.83 (10.24)	46.88 (8.92)	5.05 (–4.29 to 14.38)	.26
HR^e^ at rest (bpm)	90.58 (17.90)	89.43 (13.45)	77.67 (15.71)	84.63 (17.35)	0.69 (–14.43 to 15.80)	.92
HR at peak (bpm)	155.50 (22.18)	154.29 (21.01)	134.42 (27.54)	138.13 (27.61)	–0.98 (–16.04 to 14.09)	.89

^a^The significance level was set at *P*<.05.

^b^RER: respiratory exchange rate.

^c^Peak VO_2_: peak oxygen uptake.

^d^Peak VO_2_%pred: peak oxygen uptake percent predicted.

^e^HR: heart rate.

The CPET data in this study were obtained from only a subset of participants. As shown in Table S3 in Multimedia Appendix 2, no statistically significant differences were observed between the 2 groups across all key baseline characteristics. This indicated that although only a subset of participants completed the CPET, they constituted a representative random sample from the overall population rather than a group with systematic bias. Throughout the 8-week intervention period, no serious adverse events related to the AI-assisted telerehabilitation intervention were reported.

## Discussion

### Principal Findings

The therapeutic effects of AI-assisted remote rehabilitation training on patients with hypertension were examined in this study. This study demonstrated that AI-assisted training based on 3D skeletal pose recognition significantly improved exercise capacity and BP and increased exercise self-efficacy in patients with hypertension, superior to usual care.

### The Effect of AI-Assisted Remote Exercise Rehabilitation on Participants With Hypertension

Exercise rehabilitation for patients with hypertension is of great significance. It can not only effectively reduce BP level but also significantly improve cardiovascular function, thereby improving the quality of life for patients. However, there are some limitations in the current rehabilitation treatment of hypertension, such as the fact that patients frequently lack objective supervision and quality assurance during exercise implementation. This indicates that factors such as the specific execution methods of prescribed regimens, dosage standards, and the risk of muscle injury from nonstandardized exercises in home environments cannot be effectively safeguarded. This phenomenon not only significantly diminishes the effectiveness of interventions but also exacerbates issues related to individual variability. AI-based intelligent exercise rehabilitation is expected to solve these challenges.

In this study, the intervention group performed significantly better than the control group in terms of 6MWD, total exercise time, and peak VO_2_ for cardiopulmonary exercise testing, indicating that AI-based remote rehabilitation has a significant effect on improving exercise endurance and cardiopulmonary function. Peak VO_2_ is the core index of cardiopulmonary exercise testing, which reflects the maximum oxygen uptake capacity of patients during exercise and is an important standard to evaluate cardiopulmonary function. Although the intervention group showed significant improvement in 6MWD, the increase in peak VO₂, although statistically significant, did not reach the minimal clinically important difference, suggesting the need for longer intervention duration or a more precise exercise prescription. For objective equipment reasons, cardiopulmonary exercise testing was performed in only 24 of 62 participants before and after the intervention. CPET data were obtained from only a subset of participants. Although baseline comparisons revealed no selection bias, future research should validate these findings across the entire sample. Nevertheless, some remarkable results were obtained. This study was similar to previous studies on telerehabilitation in several ways. McDonagh et al [34,35] found that telecardiac rehabilitation and traditional rehabilitation had the same effect in restoring motor function, improving exercise capacity, and increasing physical activity; moreover, patients in the telecardiac rehabilitation group expressed higher satisfaction levels. There is some evidence to support greater adherence to telerehabilitation programs, which is particularly important in the context of health care crises (eg, due to pandemics) and in patients living in hard-to-reach, remote, and low- and middle-income areas. That said, the broader applicability of telerehabilitation in chronic disease management and remote health care still faces notable barriers—most prominently, difficulties in smartphone use among older adults [36]. As a key demographic for conditions like hypertension or heart disease, many older adult patients struggle with smartphone operation (eg, navigating apps, interpreting data feedback) due to limited digital literacy, sensory impairments, or tech unfamiliarity, which hinders engagement and risks widening health disparities. Therefore, future research should focus on assessing digital literacy’s influence on telerehabilitation response and testing tailored strategies to mitigate these barriers, ensuring remote care benefits diverse older adult populations.

Lowering BP is a core objective in hypertension clinical research. Exercise therapy, a key adjunct to pharmacotherapy, is widely proven effective for BP reduction, with the specific magnitude dependent on the exercise program design [37-40]. In this study, the remote rehabilitation (intervention) group showed a significant SBP decrease (mean reduction >12 mmHg), while no significant change was observed in DBP, a finding that is consistent with previous studies and guidelines. The ACSM and AHA recommend moderate-intensity aerobic exercise for patients with hypertension, and existing research confirms that aerobic exercise and personalized prescriptions exert a more prominent regulatory effect on SBP [41]. The lack of significant DBP reduction may relate to patients’ baseline BP, medication use, or exercise intensity. Physiologically, the significant SBP decrease can be attributed to regular exercise—particularly the “endurance and resistance” training used here—which regulates BP by improving endothelial function, reducing vascular stiffness, and mitigating oxidative stress. This aligns with findings by Guirado [42] and Kokkinos et al [43], who noted greater BP reductions with combined training. Additionally, no baseline intergroup BP differences existed, but postintervention SBP differed significantly, further validating the value of remote rehabilitation, as supported by prior research [44]. Clinically, it is meaningful that each 3 mmHg SBP reduction lowers coronary insufficiency risk by 5% to 9%, stroke risk by 8% to 14%, and all-cause mortality by 4% [45]. Notably, the impact of exercise interventions on SBP in populations with hypertension remains insufficiently understood. While exercise-induced SBP reduction is moderate but consistent, it is less pronounced than that from pharmacotherapy—although comparable to the effects of common antihypertensive drugs [37]. Future research should further explore the SBP-regulating mechanisms of exercise and assess the generalizability of such findings in real-world clinical settings.

Significant improvements in exercise self-efficacy and intervention adherence were observed with the AI-assisted remote rehabilitation program among patients with hypertension. Exercise self-efficacy is defined as an individual's confidence in their ability to successfully perform a specific physical activity. Enhanced exercise self-efficacy was achieved through multiple mechanisms. First, real-time monitoring of patient exercise data by the AI system enabled the generation of personalized feedback, allowing individuals to better understand their physiological responses and form realistic expectations about exercise outcomes. Second, a comprehensive collection of exercise demonstration videos and animated guides provided clear visual support, helping patients learn proper techniques and postures, which, in turn, boosted both skill proficiency and confidence. Third, exercise programs were dynamically adjusted using intelligent algorithms that modified intensity and difficulty based on individual health status and performance data, ensuring the intervention remained aligned with each patient’s needs and enhancing overall engagement and experience. Compared with traditional face-to-face rehabilitation programs, a remote approach demonstrates higher adherence, as patients are not required to travel to hospitals or rehabilitation centers, significantly reducing time and transportation burdens and thereby increasing participation willingness. The AI system actively prompts patients to complete daily exercise tasks, establishing a closed-loop management process of “monitoring-feedback-prompting,” which supports long-term engagement in physical activity and facilitates habit formation.

To our knowledge, this is the first randomized controlled trial to implement and evaluate an AI-assisted telerehabilitation system for patients with hypertension that utilizes real-time, AI-guided training based on 3D skeletal pose recognition. Our findings demonstrate that this technology can simultaneously and significantly improve objective exercise capacity, enhance psychological determinants of behavior such as self-efficacy, and aid in SBP control. This synergistic effect provides a new level of evidence, moving beyond remote monitoring to demonstrate the efficacy of interactive, form-correcting, and personalized exercise prescription. These results underscore the transformative potential of intelligent exercise rehabilitation for optimizing chronic disease management. Consequently, this study provides a foundation for developing individualized home-based rehabilitation programs, promoting sustained health behaviors, and advancing mobile health management models for hypertension and potentially other chronic conditions.

### Limitations

Despite the positive results of this study, there are still some limitations. First, the study sample size was relatively small and focused on the Guangdong region in China, which may limit the generalizability of the results. Future studies could expand the sample size and validate it in different regions and populations to further confirm the effectiveness of the AI-assisted telerehabilitation program. Second, the relatively short 8-week intervention period may not capture the long-term effects of AI-driven telerehabilitation on exercise capacity and cardiovascular health. Moreover, the relatively short follow-up period in this study was limited to short-term postintervention outcome assessment. Future studies could extend the follow-up period to assess the long-term effects and sustainability of the program. Finally, the lack of objective monitoring tools for participants' exercise intensity, such as using wearable devices to monitor heart rate to ensure training intensity and exercise safety, will be prioritized in future studies.

### Conclusions

This study systematically evaluated the effects of AI-based telerehabilitation on exercise ability and related health indicators of patients with hypertension through a randomized controlled trial. The results showed that the intervention had certain effects in reducing SBP and improving exercise ability and exercise self-efficacy. Moreover, the patients' compliance was high, and the intervention process was safe and feasible. As a low-cost and accessible rehabilitation training method, the AI telerehabilitation model is expected to become a convenient and effective alternative in the future.

## Data Availability

The raw data supporting the findings of this study will be made available by the corresponding author, without undue reservation.

## Appendix

Multimedia Appendix 1 CONSORT-eHEALTH checklist (V 1.6.1).

Multimedia Appendix 2 Supplementary Tables.

## Abbreviations

6MWD: 6-minute walk distance
6MWT: 6-minute walk test
ACSM: American College of Sports Medicine
AHA: American Heart Association
ANCOVA: analysis of covariance
BP: blood pressure
CONSORT: Consolidated Standards of Reporting Trials
CPET: cardiopulmonary exercise testing
DBP: diastolic blood pressure
HERB-DH1: HERB Digital Hypertension 1
MCAR: missing completely at random
PAR-Q: Physical Activity Readiness Questionnaire
peak VO2%pred: peak oxygen uptake percent predicted
peak VO2: peak oxygen uptake
SBP: systolic blood pressure
SPIRIT: Standard Protocol Items: Recommendations for Interventional Trials
WHO: World Health Organization
