# Supplementary material for: Effects of Artificial Intelligence Recognition–Based Telerehabilitation on Exercise Capacity in Patients With Hypertension: Randomized Controlled Trial
Source: J Med Internet Res. 2026 Jan 13;28:e81400. doi: 10.2196/81400 (PMC12848485; doi:10.2196/81400)
Supplement: Multimedia Appendix 2 [file jmir_v28i1e81400_app2.docx]

Supplementary Table 1. Proportion of missing data for primary and secondary outcomes

| Outcome | Total Participants, n | Missing, n | Missing, % |
| --- | --- | --- | --- |
| **Primary Outcome** |  |  |  |
| 6MWD^a^（m） | 62 | 4 | 6.5% |
| **Secondary Outcomes** |  |  |  |
| IPAQ^b^（MET-min/week） | 62 | 0 | 6.5% |
| PHQ-9^c^ | 62 | 9 | 14.5% |
| GAD-7^d^ | 62 | 9 | 14.5% |
| SF-12^e^ |  |  |  |
| PCS^f^ | 62 | 8 | 12.9% |
| MCS^g^ | 62 | 8 | 12.9% |
| Exercise self-efficacy | 62 | 8 | 12.9% |
| Weight (kg) | 62 | 0 | 0 |
| Girth (cm) | 62 | 0 | 0 |
| Hipline (cm) | 62 | 0 | 0 |
| Grip strength (kg) |  |  |  |
| Right | 62 | 5 | 8.1% |
| Left | 62 | 5 | 8.1% |
| Systolic BP (mm/Hg) | 62 | 0 | 0 |
| Diastolic BP (mm/Hg) | 62 | 0 | 0 |

^a^6MWD, the 6 Minute Walk Distance.

^b^IPAQ, International Physical Activity Questionnaire.

^c^PHQ-9, Patient Health Questionnaire-9.

^d^GAD-7, Generalized Anxiety Disorder-7.

^e^SF-12, Short-Form Health Survey 12.

^f^PCS, Physical Component Summary.

^g^MCS, Mental Component Summary.

^h^BP, Blood Pressure.

The MCAR test showed that the data were completely missing at random (χ²_596_ = 557.85, *P* =0.87).

Supplementary Table 2. Proportion of missing data for cardiopulmonary exercise test (CPET) indexes

| CPET indexes | Total Participants, n | Missing, n | Missing, % |
| --- | --- | --- | --- |
| Exercise time（s） | 24* | 3 | 12.5% |
| Maximum load（Watt） | 24 | 3 | 12.5% |
| Maximum load %pred | 24 | 3 | 12.5% |
| RER^a^ | 24 | 3 | 12.5% |
| Peak VO_2_^b^（ml/min/kg） | 24 | 3 | 12.5% |
| Peak VO_2_%pred^c^ | 24 | 3 | 12.5% |
| Anaerobic threshold（ml/min/kg） | 24 | 3 | 12.5% |
| Anaerobic threshold%pred | 24 | 3 | 12.5% |
| HR^d^ at rest（bpm） | 24 | 3 | 12.5% |
| HR at peak（bpm） | 24 | 3 | 12.5% |

^a^RER: Respiratory Exchange Rate.

^b^Peak VO_2_: Peak Oxygen Uptake.

^c^Peak VO_2_%pred: Peak Oxygen Uptake Percent Predicted.

^d^HR: Heart Rate.

Note*: The cardiopulmonary exercise test (CPET) was administered only at the one study site due to equipment availability, resulting in a planned subsample of 24 participants (12 per group) for this specific outcome. The missing rate for CPET indexes were calculated based on this subsample (24), not the entire cohort. All other outcomes were assessed for the entire cohort of 62 participants.

The MCAR test showed that the data were completely missing at random (χ²_12_ = 0, *P* > 0.99).

Supplementary Table 3. Comparison of Baseline Characteristics Between Participants With and Without Cardiopulmonary Exercise Testing (CPET) Assessment.

| Characteristic | All participants (n=62) | Participants with CPET(n=24) | Participants without CPET(n=38) | *P* |
| --- | --- | --- | --- | --- |
| Age (years), mean (SD) | 52.95 (11.46) | 50.71 (13.82) | 54.37 (9.62) | .22 |
| Gender |  |  |  | .62 |
| Male**, n (%)** | 36 (58.0) | 13 (54.2) | 23 (60.5) |  |
| Female**, n (%)** | 26 (41.9) | 11 (45.8) | 15 (39.5) |  |
| Height (m), mean (SD) | 1.64 (0.07) | 1.64 (0.05) | 1.64 (0.09) | .85 |
| Weight (kg), mean (SD) | 65.42 (13.06) | 65.37 (9.85) | 65.46 (14.86) | .98 |
| BMI^a^ (kg/m^2^), mean (SD) | 24.03 (3.35) | 24.22 (3.05) | 23.92 (3.58) | .74 |
| Education**, n (%)** |  |  |  | .93 |
| Primary and below | 4 (6.5) | 1 (4.2) | 3 (7.9) |  |
| Junior high school | 9 (14.5) | 3 (12.5) | 6 (15.8) |  |
| High school/technical secondary school | 13 (21.0) | 4 (16.7) | 9 (23.7) |  |
| College/Bachelor degree or above | 36 (58.1) | 16 (66.7) | 20 (52.6) |  |
| Smoking history**, n (%)** |  |  |  | .72 |
| Yes | 9 (14.5) | 3 (12.5) | 6 (15.8) |  |
| No | 53 (85.5) | 21 (87.5) | 32 (84.2) |  |
| Drinking history**, n (%)** |  |  |  | .12 |
| Yes | 11 (17.7) | 2 (8.3) | 9 (23.7) |  |
| No | 51 (82.3) | 22 (91.7) | 29 (76.3) |  |
| Operation history**, n (%)** |  |  |  | .33 |
| Yes | 42 (67.7) | 18 (75.0) | 24 (63.2) |  |
| No | 20 (32.3) | 6 (25.0) | 14 (36.8) |  |
| Systolic BP^b^ (mm/Hg), mean (SD) | 128.87 (12.00) | 125.96 (12.30) | 130.71 (11.60) | .13 |
| Diastolic BP (mm/Hg), mean (SD) | 85.02 (8.82) | 83.42 (9.32) | 86.03 (8.47) | .26 |
| Amount of medication, mean (SD) | 2.73 (1.57) | 2.51 (1.36) | 2.33 (1.61) | .67 |
| Number of comorbidities, mean (SD) | 1.98 (0.96) | 1.95 (0.90) | 2.00 ( 1.02) | .95 |
| Duration of hypertension (years), mean (SD) | 7.14 (7.50) | 7.67 (6.54) | 6.76 (8.23) | .17 |
| Grading of hypertension**, n (%)** |  |  |  | .08 |
| 1 | 55 (88.7) | 24 (100.0) | 31 (81.6) |  |
| 2 | 5 (8.0) | 0 (0.0) | 5 (13.2) |  |
| 3 | 2 (3.2) | 0 (0.0) | 2 (5.3) |  |
| IPAQ^c^ Physical Activity Level**, n (%)** |  |  |  | .07 |
| Low | 16 (25.81) | 10 (41.7) | 6 (15.8) |  |
| Middle | 38 (61.29) | 2 (8.3) | 6 (15.8) |  |
| High | 8 (12.90) | 12 (50.0) | 26 (68.4) |  |

^a^BMI: Body Mass Index.

^b^BP: Blood Pressure.

^c^IPAQ: International Physical Activity Questionnaire.
